# Editorial: The metabolic effect of prolactin

**DOI:** 10.3389/fendo.2023.1166172

**Published:** 2023-05-16

**Authors:** Jesper Krogh

**Affiliations:** Department of Endocrinology and Metabolism, Rigshospitalet, Copenhagen, Denmark

**Keywords:** prolactin (PRL), glucose - insulin, endothelial (dys) function, cardiovascular and cerebrovascular disease, adipocyte

Already in 1949, the Argentinian Nobel prize winner Bernado Alberto Hussey (1887-1971) showed that injection of prolactin to partially pancreatectomized dogs and cats led to high fasting blood sugar levels, glucosuria, and polyuria suggesting a diabetogenic effect of hyperprolactinemia (1). The metabolic role of prolactin is not restricted to glucose metabolism but are likely to be involved in other areas including lipid metabolism and endothelial function. This is supported by epidemiological studies finding that hyperprolactinemia is associated with increased mortality from cardiovascular disease in the background population as well as in patients with chronic kidney disease (2–4).

The association between prolactin and glucose metabolism is explored in the review ‘*Metabolic effects of prolactin*’by Pirchio et al. A strong indication of prolactin’s role in the glucoinsulinemic profile is that prolactin receptors are present on rat beta-cells and their expression is increased during pregnancy. This is supported by *in vitro* studies showing that isolated pancreatic islets from rats demonstrate a raise in insulin secretion and increase in beta-cell mass in response to prolactin exposure. In human subjects, chronic hyperprolactinemia is associated with postprandial hyperinsulinemia and exaggerated insulin secretory response to glucose. Normalization of prolactin levels in patients with hyperprolactinemia by either surgery or treatment with dopamine agonists results in normalization of glucose and insulin levels. Larger cohort studies of subjects with prolactin levels within the reference limits finds that both low as well as high prolactin levels are associated with the risk of diabetes mellitus. The association between prolactin and gluco-insulinemic profile is therefore likely to be a u-curve, with low prolactin as well as hyperprolactinemia producing a diabetic state. An obvious question is how high (or low) prolactin levels should be to have diabetogenic effects. The authors discuss a previously published concept of HomeoFIT-PRL that suggests that transiently elevated prolactin levels between 25 and 100 μg/L in the absence of other pathophysiological causes represent physiologic adaptation to increased metabolic demands. They speculate that future studies of metabolic effects of prolactin within this range may lead to changes in recommendations for treatment of hyperprolactinemia, such as not to necessarily reduce prolactin to normal or below normal reference levels.

A previous report on the association between chronic kidney disease and prolactin found that higher prolactin was independently associated with reduced flow-mediated dilation and increased pulse wave velocity suggesting a role for prolactin on blood vessels (5). In the review ‘*The interplay of prolactin and cardiovascular disease*’ by Glezer et al. the authors argue that prolactin is associated with increased risk for cardiovascular disease through its effect on, amongst other things, endothelial function. It has been shown that prolactin stimulates angiogenesis by promoting endothelial cell proliferation or by upregulation pro-angiogenic factors and alter endothelial function by reducing nitrogen oxide leading. The above factors may contribute to the observed reduced flow-mediated dilation in patients with prolactinomas. In addition, the intima-thickness has been reported increased in patients with prolactinomas and in one study the intima-thickness was reduced after 6 months of treatment with bromocriptine and overall, it appears that prolactin may play a role on endothelial function which needs to be confirmed in future studies.

Several uncontrolled studies reports that treatment with dopamine agonists in patients with prolactinomas reduces weight, waist circumference, c reactive protein, hba1c, and triglycerides (6). However, as reported in the review by Kirsch et al. ‘*Metabolic effects of prolactin and the role of dopamine antagonists*’ these studies do not allow any causal inference on the metabolic effects of prolactin but may suggest an independent metabolic role of dopamine agonists. The fact that dopamine 2 receptors are present in adipose tissue and that the above-mentioned studies rarely identify any association between change in prolactin and change in metabolic variables makes the authors suggest that dopamine agonists may have an independent positive effect on metabolic variables. This is supported by a recent study finding that pituitary surgery of prolactinoma patients decreased cholesterol and triglycerides whereas cabergoline treatment reduced LDL as well as glucose levels (7).

While antipsychotic induced hyperprolactinemia is reported to affect up to 70% of patients with schizophrenia treated with prolactin enhancing antipsychotics this receives little attention among traditional endocrinologist (8). A recent Finnish nationwide study suggests that the use of prolactin enhancing antipsychotics increases the risk of breast cancer (9), which highlights the importance of this topic. An original study by Zhu et al. reports in ‘*Integrating machine learning with electronic health record data to facilitate detection of prolactin levels and pharmocovigilance in olanzapine-treated patients*’ the use of machine learning including 368 clinical and biochemical factors collected from electronic patient files to predict prolactin values. The analysis identified 15 factors associated which influenced prolactin levels, with female gender, concomitant use of risperidone and sulpride being associated with high prolactin levels, which is in concordance with existing knowledge. Also, factors such creatinine kinase and sodium levels were identified as having an impact on prolactin levels. The application of machine learning for prediction of prolactin response to antipsychotic is novel and deserves further exploration.

In summary, data suggest that prolactin is involved in glucose metabolism, lipid metabolism, endothelial function, obesity, and may even be associated with increased cardiovascular mortality. However, several issues need to be clarified in order to advance our knowledge in this field: gender affects prolactin levels, and it appears that gender may affect the association to cardiovascular mortality (2) and therefore the interaction between gender/estrogen and the association between metabolic factors and prolactin should be further explored. Also, the apparent u-curve observed for prolactin levels and glucose metabolism is not evident or not described in the association between prolactin and lipid metabolism, weight, or endothelial dysfunction. To provide clinical evidence for treating prolactin for metabolic reasons, we need to be able to test our hypothesis in human models. However, dopamine agonists have positive metabolic properties themselves, and the most potent prolactin enhancing drugs in the form of antipsychotics also have adverse metabolic profiles making it difficult to entangle the effect of prolactin as well as the effects of ‘normal’ 23-kDa prolactin opposed to the post-proteolytic peptides, e.g. 16-kDa prolactin (10). Prolactin for injection in humans or prolactin receptor blockers are not yet commercially available for human subjects, which would allow us to explore the metabolic effects of prolactin in controlled studies.

## Author contributions

The author confirms being the sole contributor of this work and has approved it for publication.
